# Dietary polyphenols influence antimetabolite agents: methotrexate, 6-mercaptopurine and 5-fluorouracil in leukemia cell lines

**DOI:** 10.18632/oncotarget.20501

**Published:** 2017-08-24

**Authors:** Amani Mahbub, Christine Le Maitre, Sarah Haywood-Small, Neil Cross, Nicola Jordan-Mahy

**Affiliations:** ^1^ Laboratory Medicine College, Pathology Department, Umm Al Qura University, Makkah, Saudi Arabia; ^2^ Biomolecular Sciences Research Center, Sheffield Hallam University, Sheffield, UK

**Keywords:** leukemia, 6-mercaptopurine, polyphenols, methotrexate, 5-fluorouracil

## Abstract

Polyphenols have been previously shown to sensitize leukemia cell lines to topoisomerase inhibitors. Here, we assess the effects of five polyphenols when used alone and in combination with antimetabolites: methotrexate, 6-mercaptopurine and 5-fluorouracil; in lymphoid and myeloid leukemia cells lines, and non-tumor control cells. The effects of combined treatments were investigated on ATP and glutathione levels, cell-cycle progression, DNA damage and apoptosis.

Polyphenols antagonized methotrexate and 6-mercaptopurine induced cell-cycle arrest and apoptosis in most leukemia cell lines. This was associated with reduced DNA damage and increased glutathione levels, greater than that seen following individual treatments alone.

In contrast, 5-fluorouracil when combined with quercetin, apigenin and rhein caused synergistic decrease in ATP levels, induction of cell-cycle arrest and apoptosis in some leukemia cell lines. However, antagonistic effects were observed when 5-fluorouracil was combined with rhein and *cis*-stilbene in myeloid cell lines. The effects were dependant on polyphenol type and chemotherapy agent investigated, and cell type treated. Interestingly treatment of non-tumor control cells with polyphenols protected cells from antimetabolite treatments.

This suggests that polyphenols modulate the action of antimetabolite agents; more importantly they antagonized methotrexate and 6-mercaptopurine actions, thus suggesting the requirement of polyphenol-exclusion during their use.

## INTRODUCTION

The mortality of leukemia is still high despite considerable improvements in tolerance and efficacy of chemotherapeutic agents [1]. Antimetabolite agents such as methotrexate, 6-mercaptopurine and 5-fluorouracil are some of the most commonly used chemotherapy agents used to treat leukemia [1]. They have National Institute for Health and Care Excellence and Food and Drug Administration approval for their therapeutic and maintenance use in leukemia [2, 3]. Methotrexate is used in the treatment of all types of leukemia; and commonly used in the treatment of childhood acute lymphoid leukemia [1, 4–6]. 6-Mercaptopurine is approved for acute lymphocytic leukemia, childhood acute lymphoid leukemia, as well as acute and chronic myeloid leukemia [1, 4–5]; whilst, 5-fluorouracil is used primarily in the treatment of acute myeloid leukemia [7–8]. However, these antimetabolites are non-specific, affecting both normal and malignant cells. As a consequence, these drugs are associated with severe side-effects and drug resistance [1–5, 9].

Epidemiological data has shown that diets rich in polyphenols particularly from fruits and vegetables [10] significantly improve quality of life [11] and survival rates of patients with chronic diseases, including cancer [12]. Polyphenols possess a broad range of biological properties in cancer cells and have been shown to inhibit cell proliferation; arrest cell-cycle and inhibit several protein kinases and induce apoptosis [13, 14]. Our earlier work demonstrated that polyphenols reduced ATP levels, caused cell-cycle arrest and induced apoptosis, particularly in lymphoid leukemia cells; with limited effects seen in non-tumor hematopoietic control cells [15].

In solid tumors polyphenols such as quercetin, have been shown to synergistically induce apoptosis when used in combination with chemotherapy agents such as: cisplatin, doxorubicin, and 5-fluorouracil [16–19]. Similarly, our work in leukemia cell lines showed that quercetin, apigenin, emodin, and *cis*-stilbene, enhanced the action of topoisomerase inhibitors: etoposide and doxorubicin; synergistically enhancing their pro-apoptotic effects; whilst protecting non-tumor control cells [20–21]. These polyphenols are representative of the major polyphenol groups and are commonly found in diet. Quercetin (a flavonol) and apigenin (a flavone) are classified as flavonoids and commonly found in fruits (apples and blueberries), vegetables (onions, broccoli, parsley, celery, and rhubarb) nuts, seeds, flowers and teas [22–23]. Emodin and rhein are classified as anthraquinones and found in rhubarb [24], herbs (senna, purlane and aloe), peas, cabbage, lettuce and beans [25]; whilst, cis-stilbene is classified as a stilbene and is commonly found in grapes, peanuts [11] and rhubarb [23].

Our earlier findings have led to the present investigation examining the effects of these five polyphenols (quercetin, apigenin, emodin, rhein, and *cis*-stilbene) on the action of anti-metabolic drugs: methotrexate; 6-mercaptopurine and 5-fluorouracil in leukemia cell lines.

## RESULTS

### Effects of antimetabolite agents alone on ATP levels and caspase 3 activity in leukemia cell lines following 24 hrs treatment

**Figure 1:**
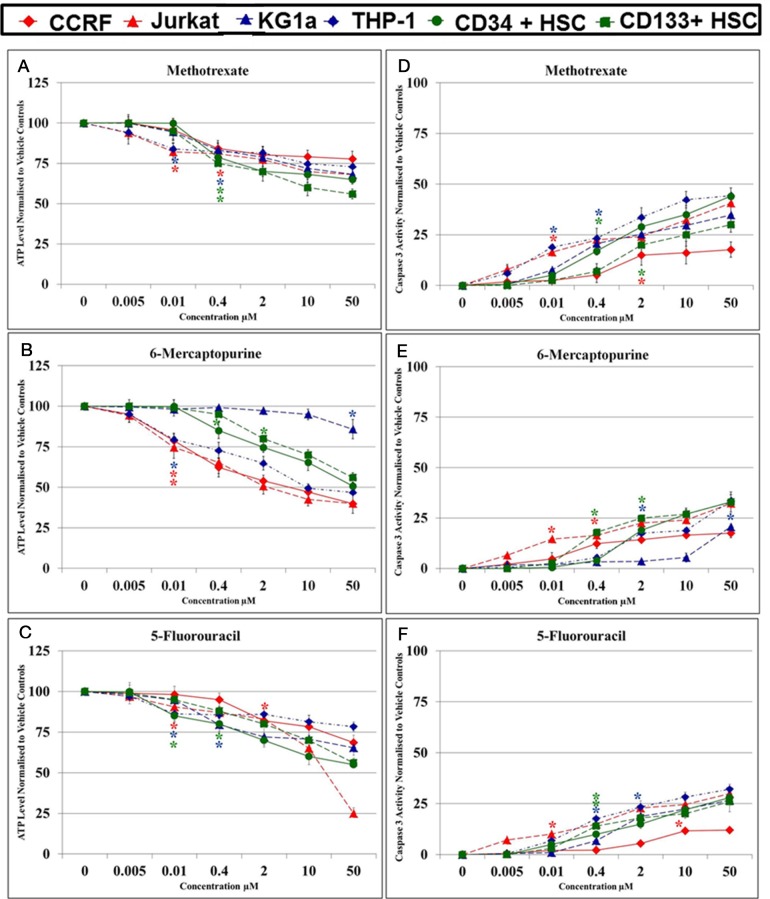
Effect of methotrexate, 6-mercaptopurine and 5-fluorouracil alone on ATP levels **(A, B & C)** and caspase 3 activity **(D, E & F)** in two lymphoid (CCRF-CEM and Jurkat), two myeloid (THP-1 and KG-1a) leukemia cell lines, and two non-tumor control cells (CD133^+^ HSC and CD34^+^ HSC). The lowest significant doses (LSD): which caused a significant reduction on ATP levels and induction of caspase 3 activity when compared to the vehicle control were determined for each anti-metabolite agent in each cell lines. The ^*^ indicated for LSD in each cell line.

Methotrexate, 6-mercaptopurine and 5-fluorouracil induced a dose dependant decrease in ATP levels (Figure 1). The lowest significant doses (LSD) and IC_50_ doses for methotrexate, 6-mercaptopurine and 5-fluorouracil alone, which reduced ATP levels in comparison to the vehicle control, varied depending on the cell lineage, and are summarised in Table 1. Treatment doses between 0.01 μM and 2 μM were shown to significantly reduce ATP levels after 24 hrs treatment in lymphoid leukemia cell lines and non-tumor control cells (P≤0.05). The myeloid leukemia cell lines were more resistant to antimetabolite agents requiring treatment doses between 0.01 μM and 50 μM to significantly reduce ATP levels at 24 hrs (P≤0.05). The highest doses used were in the KG1a cell line which required a 50 μM 6-mercaptopurine treatment for 24 hrs to significantly reduce ATP levels (P≤0.05). The LSDs for the induction of caspase 3 activity for methotrexate, mercaptopurine and 5-fluorouracil followed a similar pattern to the LSDs for ATP levels (Table 1). Once again, the lymphoid leukemia cells and non-tumor control cells were more sensitive to antimetabolite treatments, whilst the myeloid cells, particularly the KG1a cells were more resistant. The LSDs for caspase-3 activity were found to be the same or slightly higher than the LSDs for ATP levels, as a reflection of the progression from a reduction of cell viability (measured as ATP levels) to early stage apoptosis (Table 1). These LSDs were used to investigate the effects of combination treatments and establish whether polyphenols have synergistic or antagonistic effects on antimetabolite agents.

**Table 1:** The lowest significant dose (LSDs) and IC_50_ Doses: of methotrexate (MTX), 6-mercaptopurine (6-MP) and 5-fluorouracil (5-FLU) which reduced ATP levels and increased caspase 3 activity (CASP 3) when compared to the vehicle control (P≤0.05) in two lymphoid (Jurkat and CCRF-CEM) and two myeloid (THP1 and KG-1a) leukemia cell lines; and two non-tumor control hematopoietic stem cells (CD34^+^ HSCs and CD133^+^ HSCs). The LSDs for ATP levels where used in subsequent investigation of combination treatments on cell-cycle progression, ATP and glutathione levels and DNA damage. The LSDs for caspase 3 activity were used for further investigation of apoptosis

Cell Type		Doses	MTX		6-MP		5-FLU	
			ATP	CASP 3	ATP	CASP 3	ATP	CASP 3
Lymphoid Cells	Jurkat	LSD	0.01μM	0.01μM	0.01μM	0.01μM	0.01μM	0.01μM
		IC_50_	>50 μM	>50 μM	2 μM	>50 μM	30 μM	>50 μM
	CCRF-CEM	LSD	0.4μM	2μM	0.01μM	0.4μM	2μM	10μM
		IC_50_	>50 μM	>50 μM	6 μM	>50 μM	>50 μM	>50 μM
Myeloid Cells	THP1	LSD	0.01μM	0.01μM	0.01μM	2μM	0.01μM	0.4μM
		IC_50_	>50 μM	>50 μM	10 μM	>50 μM	>50 μM	>50 μM
	KG1a	LSD	0.4 μM	0.4 μM	50μM	50μM	0.4μM	2μM
		IC_50_	>50 μM	>50 μM	>50 μM	>50 μM	>50 μM	>50 μM
Non-Tumor Control Cells	CD34^+^ HSCs	LSD	0.4 μM	0.4 μM	0.4 μM	2μM	0.01μM	0.4μM
		IC_50_	>50 μM	>50 μM	50 μM	>50 μM	>50 μM	>50 μM
	CD133^+^ HSCs	LSD	0.4 μM	2 μM	2μM	0.4 μM	0.4 μM	0.4μM
		IC_50_	>50 μM	>50 μM	>50 μM	>50 μM	>50 μM	>50 μM

### Combination effects of antimetabolites and polyphenols on ATP levels in leukemia cell lines and non-tumor cells at 24 hrs

**Figure 2:**
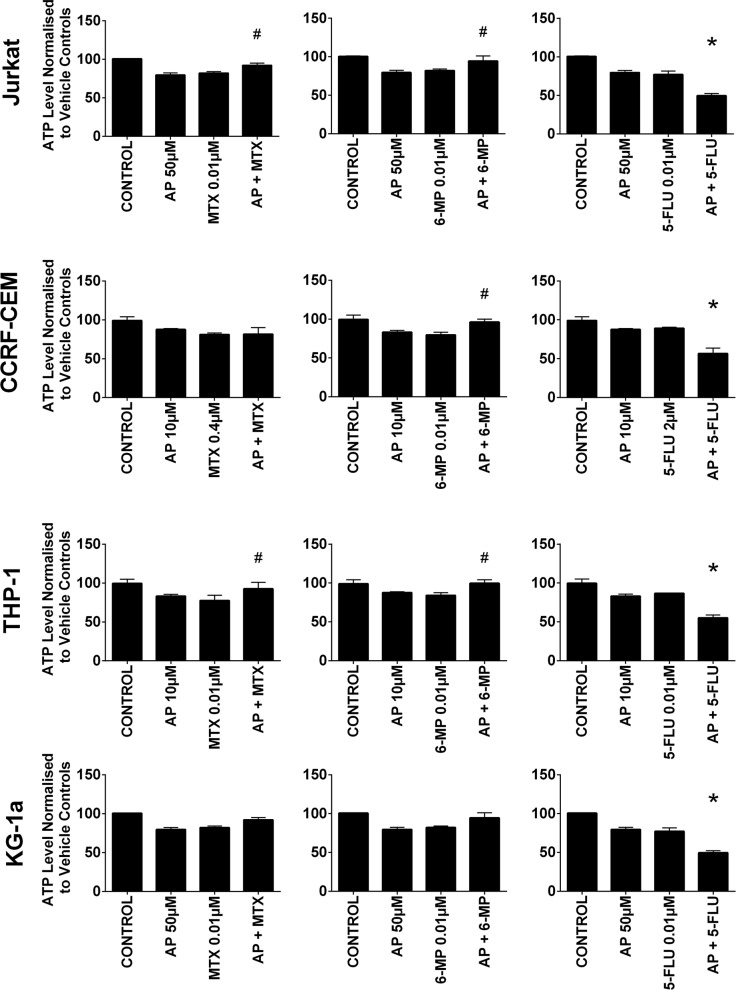
The effect of methotrexate (MTX), 6-mercaptopurine (6-MP) and 5-fluorouracil (5-FLU) when used in combination with apigenin (AP) on ATP levels: in two lymphoid (Jurkat and CCRF-CEM) and two myeloid (THP-1 and KG-1a) leukemia cell lines, evaluated by CellTiter-Glo® assay Cells were treated with MTX, 6-MP or 5-FLU and apigenin alone and in combination for 24 hr using their lowest-significant doses (LSD); together with a vehicle control. All data was normalised to the vehicle control which was assigned 100% cell viability. The data was expressed as medians and ranges (n=4). Effects of combination treatments were statistically classified as synergistic (^*^) causing a decrease in ATP levels or antagonistic (^#^) causing an increase in ATP levels; when compared to vehicle control, drugs alone and expected values of combination treatments. Statistical significant was set at P≤0.05.

Treatment of all leukemia cell lines with methotrexate, 6-mercaptopurine and 5-fluorouracil alone caused a significant decrease in ATP levels (Supplementary Figure 1). However, the effect of these chemotherapy agents was greatly affected by the action of polyphenols during combination treatment. Most notably apigenin was shown to have antagonistic effects of 6-mercaptopurine and methotrexate (Figure 2). When apigenin was combined with 6- mercaptopurine; it caused a significant increase in ATP level in both lymphoid cell lines and one myeloid cell line (THP-1) (P≤0.05) (Figure 2). Similarly, when combined with methotrexate, it increased ATP levels in one lymphoid (Jurkat) and one myeloid (THP-1) cell line (P≤0.05). In contrast when apigenin was combined with 5-fluorouracil, there was a synergistic effect and a decrease in ATP levels in all leukemia cells lines (P≤0.05).

**Figure 3:**
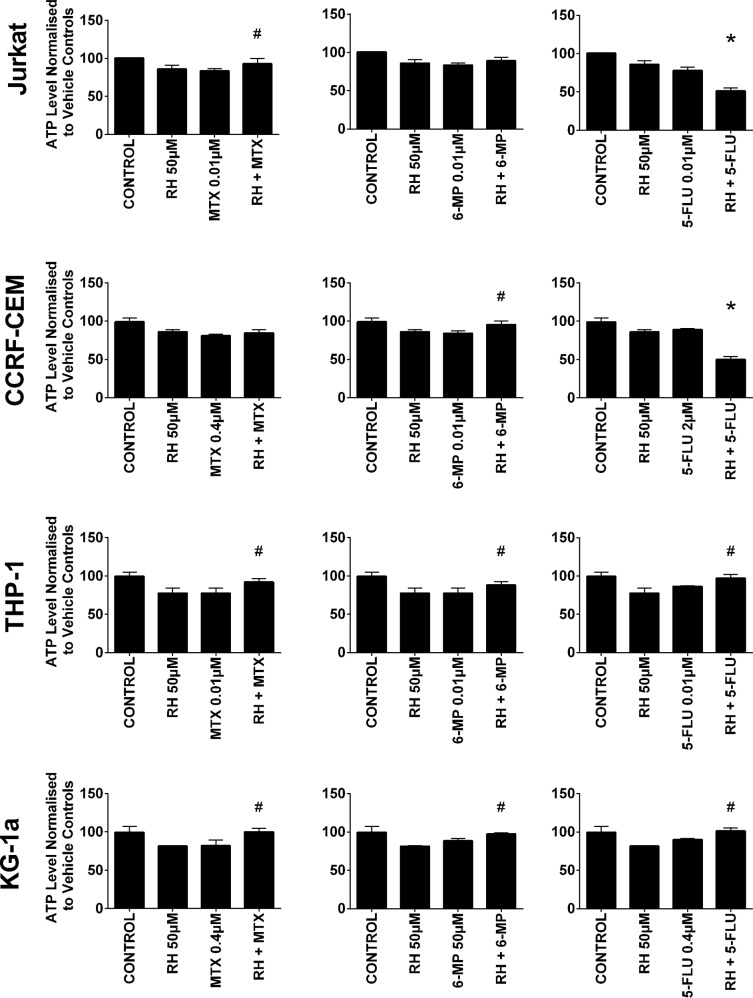
The effect of methotrexate (MTX), 6-mercaptopurine (6-MP) and 5-fluorouracil (5-FLU) when used in combination with rhein (RH) on ATP levels: in two lymphoid (Jurkat and CCRF-CEM) and two myeloid (THP-1 and KG-1a) leukemia cell lines This was evaluated by CellTiter-Glo® assay. Cells were treated with MTX, 6-MP or 5-FLU and rhein alone and in combination for 24 hr using their lowest-significant doses (LSD); together with a vehicle control. All data was normalised to the vehicle control which was assigned 100% cell viability. The data was expressed as medians and ranges (n=4). Effects of combination treatments were statistically classified as synergistic (^*^) causing a decrease in ATP levels or antagonistic (^#^) causing an increase in ATP levels; when compared to vehicle control, drugs alone and expected values of combination treatments. Statistical significant was set at P≤0.05.

Rhein was also shown to antagonize the action of 6-mercaptopurine and methotrexate (Figure 3). Rhein when used in combination with 6-mercaptopurine, induced an increase in ATP levels in one lymphoid cell line (CCRF-CEM) and both myeloid cell lines (P≤0.05). A similar antagonistic effect was seen when rhein was combined with methotrexate, where a significant increase in ATP levels was seen in one lymphoid cell line (Jurkat) and both myeloid cell lines (P≤0.05) (Figure 3). In contrast when rhein was used in combination with 5-fluorouracil there was a synergistic decrease in ATP levels in the lymphoid cell lines; but an antagonistic effect in the myeloid cell lines (P≤0.05).

Antagonistic effects were also seen when emodin was combined with methotrexate in the Jurkat lymphoid cell line and both myeloid cell lines (P≤0.05), and when emodin was combined with 6-mercaptopurine in both lymphoid cell lines and the THP-1 myeloid cell line (P≤0.05) (Supplementary Figure 1). The only interactions seen with quercetin were in the KG1a cell line, where methotrexate was antagonized by quercetin and ATP levels were increased (P≤0.05). In contrast when quercetin was used in combination with 5-fluorouracil there was a synergistic reduction in ATP levels (P≤0.05) (Supplementary Figure 1). Finally *cis*-stilbene was shown to antagonize the action of all three antimetabolites. It antagonized the action of methotrexate in Jurkat and THP-1 cell lines; 6-mercaptopurine in Jurkat, CCRF-CEM, and THP-1 cell lines and 5-fluorouracil in THP-1 and KG-1a cell lines (P≤0.05).

In non-tumor control cells (CD34^+^HSC and CD133^+^HSC), all studied polyphenols when used in combination with each of the three antimetabolite agents antagonized their effects significantly increasing ATP levels and cell survival (P≤0.05) (Supplementary Figure 1).

### Combination effects of antimetabolites and polyphenols on apoptosis

**Figure 4:**
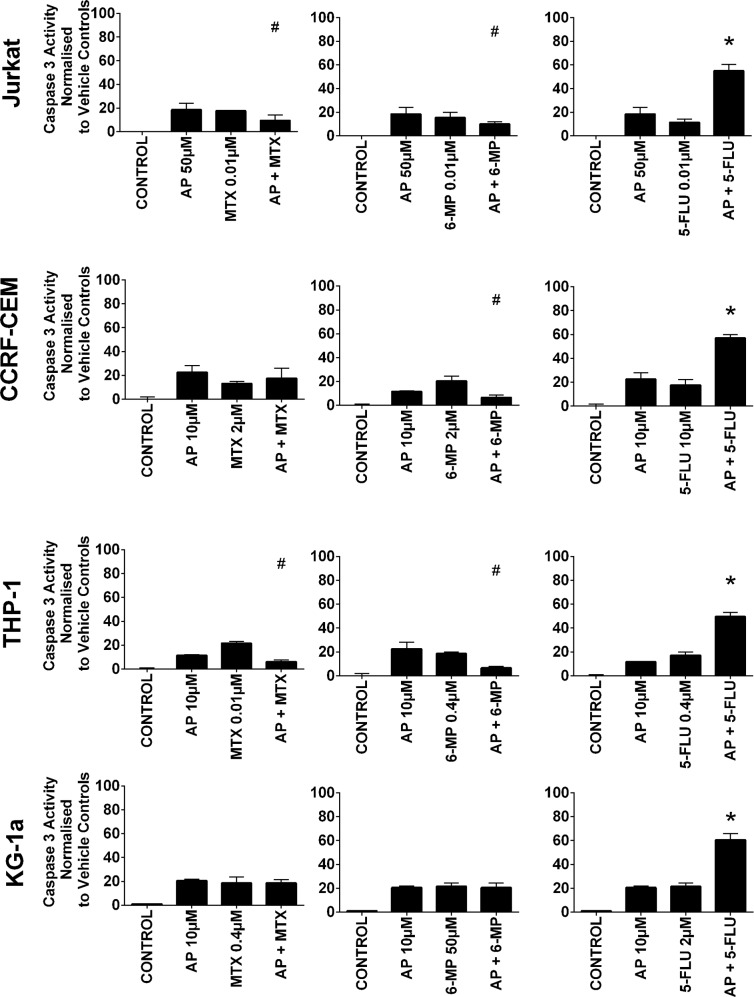
The effect of methotrexate (MTX), 6-mercaptopurine (6-MP) and 5-fluorouracil (5-FLU) when used in combination with apigenin (AP) on caspase 3 activity: in two lymphoid (Jurkat and CCRF-CEM) and two myeloid (THP-1 and KG-1a) leukemia cell lines This was evaluated by NucView caspase 3 activity assay. Cells were treated with MTX, 6-MP or 5-FLU and apigenin alone and in combination for 24 hr using their lowest-significant doses (LSD); together with a vehicle control. All data was normalised to the vehicle control which was assigned 0% apoptosis. The data was expressed as medians and ranges (n=4). Effects of combination treatments were statistically classified as synergistic (^*^) causing an increase in caspase 3 activity or antagonistic (^#^) causing an decrease in caspase 3 activity; when compared to vehicle control, drugs alone and expected values of combination treatments. Statistical significant was set at P≤0.05.

**Figure 5:**
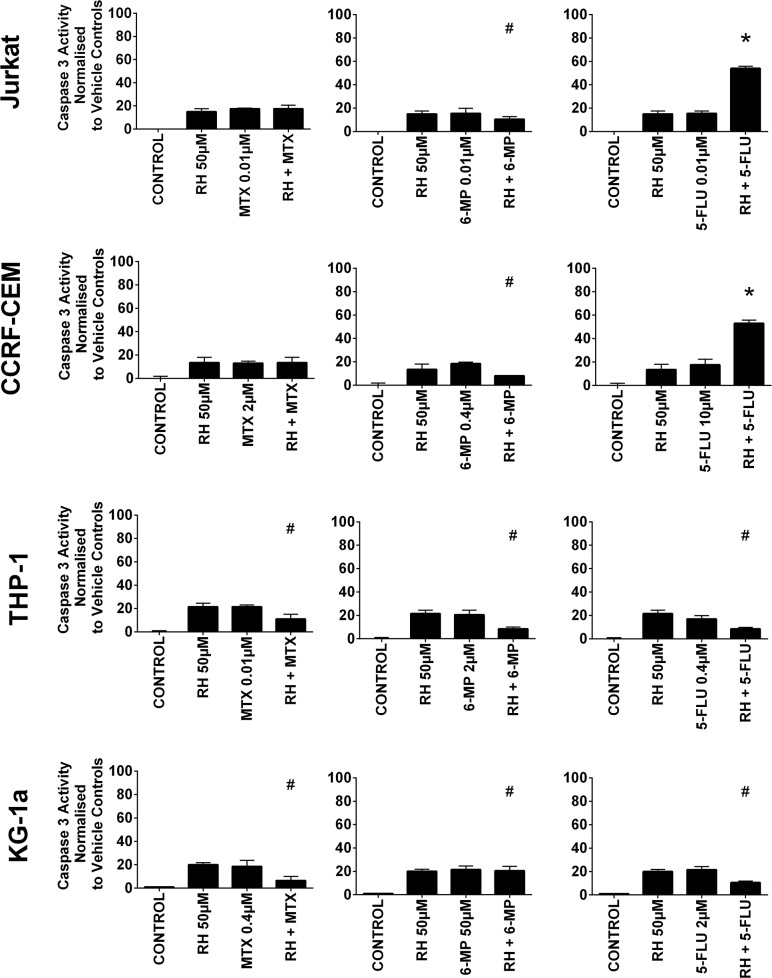
The effect of methotrexate (MTX), 6-mercaptopurine (6-MP) and 5-fluorouracil (5-FLU) when used in combination with rhein (RH) on caspase 3 activity: in two lymphoid (Jurkat and CCRF-CEM) and two myeloid (THP-1 and KG-1a) leukemia cell lines This was evaluated by NucView caspase 3 activity assay. Cells were treated with MTX, 6-MP or 5-FLU and rhein alone and in combination for 24 hr using their lowest-significant doses (LSD); together with a vehicle control. All data was normalised to the vehicle control which was assigned 0% apoptosis. The data was expressed as medians and ranges (n=4). Effects of combination treatments were statistically classified as synergistic (^*^) causing an increase in caspase 3 activity or antagonistic (^#^) causing an decrease in caspase 3 activity; when compared to vehicle control, drugs alone and expected values of combination treatments. Statistical significant was set at P≤0.05.

All three antimetabolite agents significantly induced caspase 3 activity in all leukemia cell lines (P≤0.05) (Figure 4 & 5). Consistent with our observations of ATP levels; apigenin was shown once again to antagonize the action of methotrexate and 6-mercaptopurine. Apigenin when used in combination with methotrexate significantly decreased caspase 3 activity in one lymphoid (Jurkat) and one myeloid (THP-1) cell line (P≤0.05) (Figure 4). Apigenin also antagonized the action of 6-mercaptopurine in both lymphoid and one myeloid (THP-1) cell line resulting in a decrease in caspase 3 activity (P≤0.05) (Figure 4). A synergistic effect was however seen in all leukemia cell lines, when apigenin was combined with 5-fluorouracil; here caspase 3 activity was significantly increased (P≤0.05) (Figure 4).

Rhein was also shown to antagonize the pro-apoptotic effect of 6-mercaptopurine in all leukemia cell lines, significantly decreasing caspase 3 activity (P≤0.05) (Figure 5). Likewise when rhein was combined with methotrexate or 5-fluorouracil in the myeloid cell lines caspase 3 activity was also antagonized (P≤0.05) (Figure 5). However, when rhein was combined with 5-fluorouracil in the lymphoid cell lines there was a synergistic increase in caspase 3 activity (P≤0.05) (Figure 5).

A synergistic increase in caspase 3 activity was also seen when quercetin was combined with 5-fluorouracil in KG-1a cells (P≤0.05) (Supplementary Figure 2). Emodin however was shown to antagonize the pro-apoptotic effect of methotrexate and 6-mercaptopurine in three out of four leukemia cell lines (P≤0.05) (Supplementary Figure 2). *Cis*-stilbene antagonized the pro-apoptotic effect of all three antimetabolite agents; however, its effect was dependent on cell lineage. *Cis*-stilbene antagonized 6-mercaptopurine induced caspase 3 activity in both lymphoid cell lines and methotrexate induced caspase 3 activity in one cell line (CCRF-CEM) (P≤0.05) (Supplementary Figure 2). In the myeloid cell lines, *cis*-stilbene antagonized the pro-apoptotic effect of all three antimetabolite agents, decreasing caspase 3 activity (Supplementary Figure 2). In non-tumor control cells, all three antimetabolite agents were antagonized by all five polyphenols decreasing antimetabolite induction of caspase 3 (P≤0.05) (Supplementary Figure 2). These effects were confirmed by morphological assessment of apoptosis (Supplementary Figure 3).

### The effect of combination treatments on cell-cycle progression

**Figure 6:**
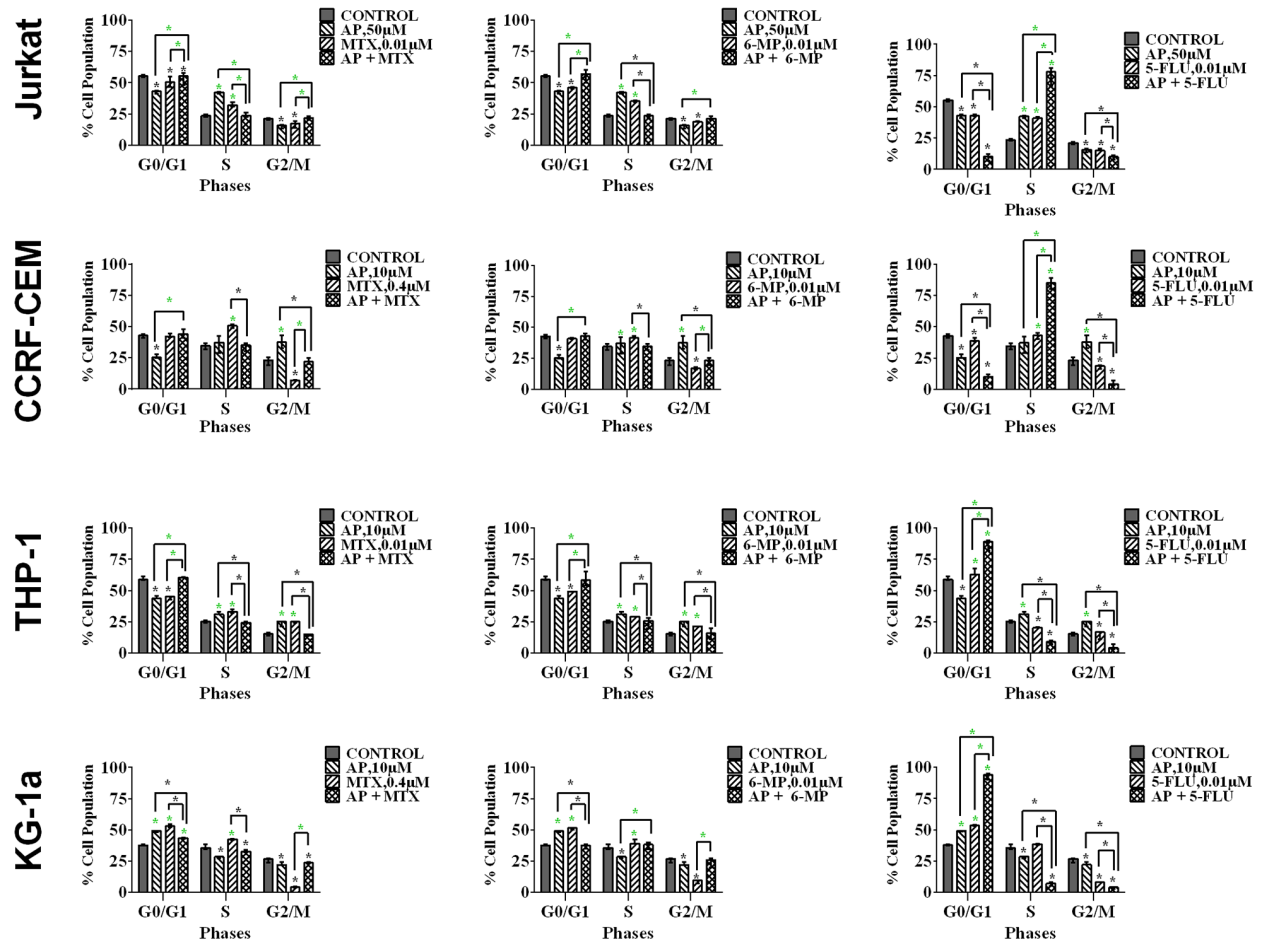
The effect of methotrexate (MTX), 6-mercaptopurine (6-MP) and 5-fluorouracil (5-FLU) on cell cycle progression, when used in combination with apigenin (AP): in two lymphoid leukemia cell lines (Jurkat and CCRF-CEM) and two myeloid leukemia cell lines (THP-1 and KG-1a) This was analysed by flow cytometry following propidium iodide staining. Cells were treated with MTX, 6-MP or 5-FLU and apigenin alone and in combination for 24 hrs using their lowest-significant doses (LSD) as determined by CellTiter-Glo assay, together with a vehicle control. The percentage of cells in each phase was analysed with FlowJo software using Waston pragmatic model. The data was expressed as medians with ranges (n=4). Statistical significance of combination treatments were determined and compared with the vehicle control and the individual treatments alone. The green asterisk (^*^) represents significant increase in cell accumulation in a phase of the cell cycle; whilst the black asterisk (^*^) indicates a significant decrease in cell accumulation in a phase of the cell cycle. Statistical significant was set at P≤0.05.

**Figure 7:**
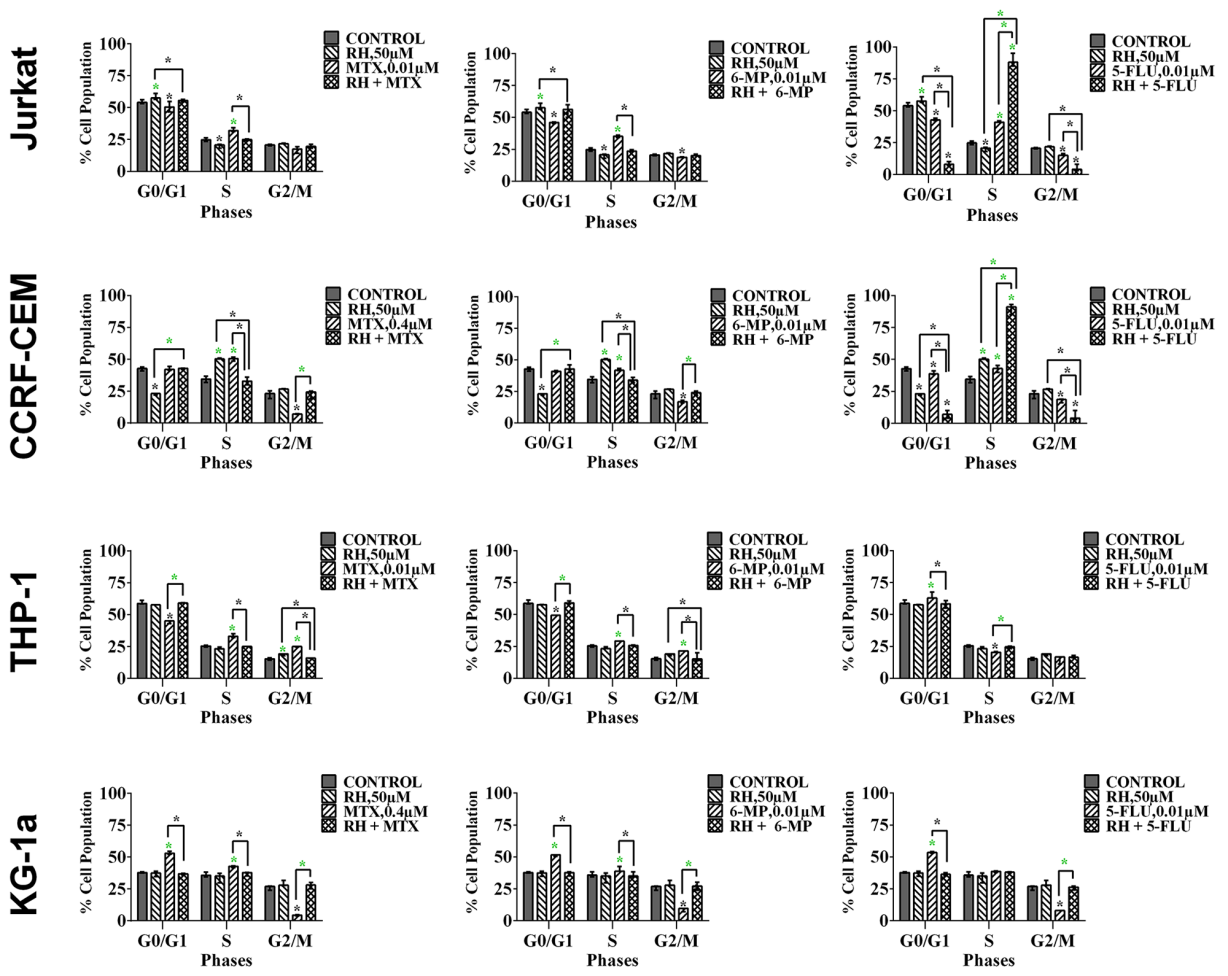
The effect of methotrexate (MTX), 6-mercaptopurine (6-MP) and 5-fluorouracil (5-FLU) on cell cycle progression, when used in combination with rhein (RH): in two lymphoid leukemia cell lines (Jurkat and CCRF-CEM) and two myeloid leukemia cell lines (THP-1 and KG-1a) This was analysed by flow cytometry following propidium iodide staining. Cells were treated with MTX, 6-MP or 5-FLU and rhein alone and in combination for 24 hrs using their lowest-significant doses (LSD) as determined by CellTiter-Glo assay, together with a vehicle control. The percentage of cells in each phase was analysed with FlowJo software using Waston pragmatic model. The data was expressed as medians with ranges (n=4). Statistical significance of combination treatments were determined and compared with the vehicle control and the individual treatments alone. The green asterisk (^*^) represents significant increase in cell accumulation in a phase of the cell cycle; whilst the black asterisk (^*^) indicates a significant decrease in cell accumulation in a phase of the cell cycle. Statistical significant was set at P≤0.05.

Methotrexate and 6-mercaptopurine alone caused S-phase cell cycle arrest in all leukemia cell lines (P≤0.05). This was antagonized by all the polyphenols (data not shown); the most significant effects were seen following combination with apigenin (Figure 6 & Supplementary Figure 4) and rhein (Figure 7), in all leukemia cell lines (P≤0.05). 5-fluorouracil alone caused S-phase arrest in lymphoid cell lines and G_0_G_1_ arrest in the myeloid cells (P≤0.05) (Figure 6). Apigenin when combined 5-fluorouracil caused a synergistic increase in cells accumulating in S-phase in both lymphoid cell lines (P≤0.05), and G_0_G_1_-phase in myeloid cells (P≤0.05) (Figure 6 and Supplementary Figure 4). Rhein also acted synergistically with 5-fluorouracil causing S-phase arrest in the lymphoid cell lines cells (P≤0.05) (Figure 7).

### The effect of combination treatments on cell glutathione levels

**Figure 8:**
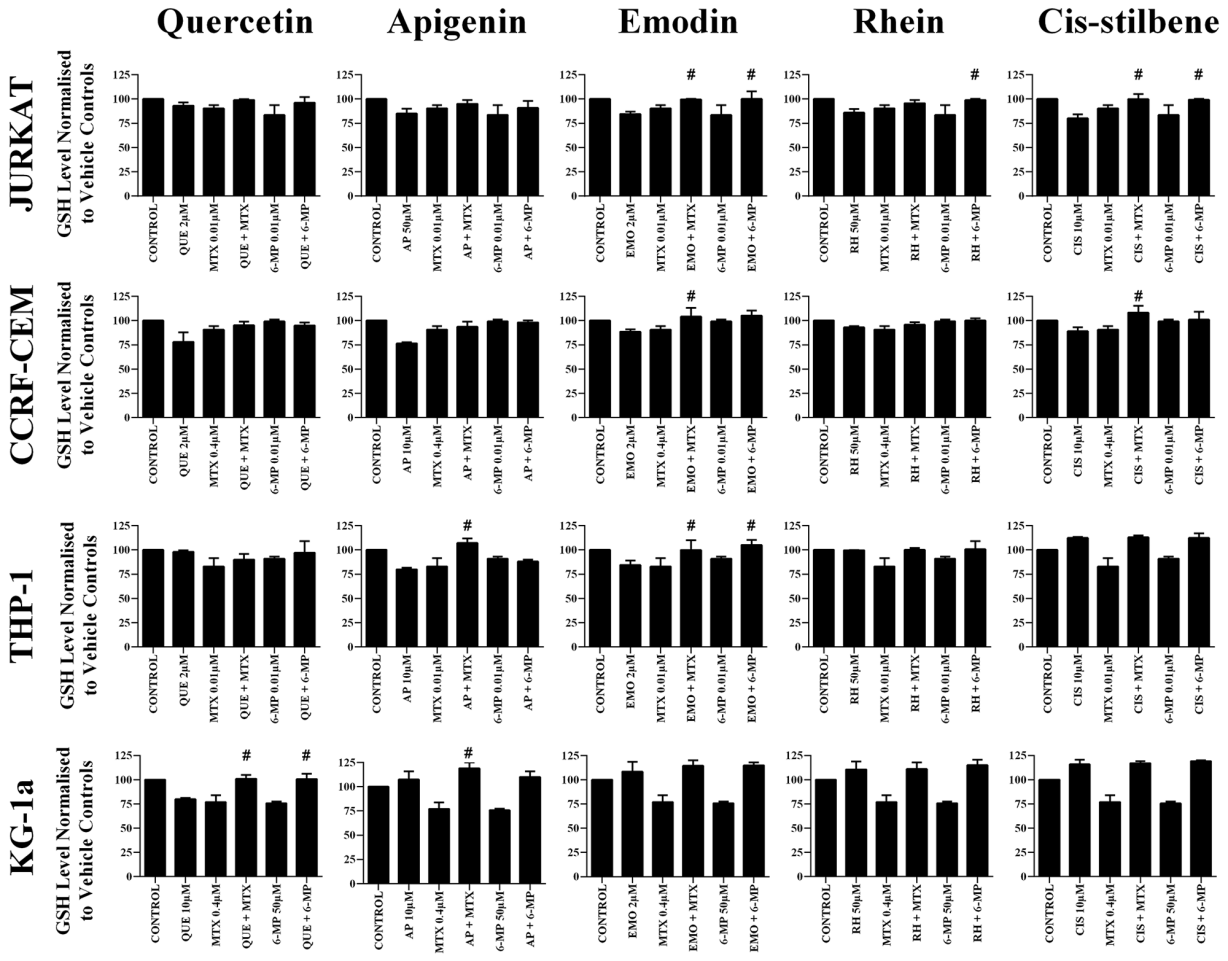
The effect of methotrexate (MTX) and 6-mercaptopurine (6-MP) when used in combination with quercetin (QUE), apigenin (AP), emodin (EMO), rhein, (RH) or cis-stilbene (CIS) on glutathione (GSH) levels: in two lymphoid (Jurkat and CCRF-CEM) and two myeloid (THP-1 and KG-1a) leukemia cell lines GSH levels were evaluated by the GSH-Glo™ Glutathione. Cells were treated with MTX or 6-MP and polyphenols alone and in combination for 24 hrs using their lowest-significant doses (LSD). Data was normalised to the vehicle control which was assigned 100% of GSH levels. The data was expressed as medians and ranges (n=4). Effects of combination treatments were statistically classified as synergistic (^*^) causing a decrease in GSH levels or antagonistic (^#^) causing an increase in GSH levels; when compared to vehicle control, drugs alone and expected values of combination treatments. Statistical significant was set at P≤0.05.

Methotrexate and 6-mercaptopurine alone caused a decrease in glutathione levels in all leukemia cell lines (P≤0.05) (Figure 8). However, these reductions were antagonized by combination treatment with each polyphenol; resulting in an overall elevation in glutathione levels which were comparable, or higher than untreated leukemia cells or non-tumor control cells (P≤0.05) (Figure 8). These results were confirmed using CMFDA staining (Supplementary Figure 5), and were found to follow the same trends shown by the GSH-Glo™ glutathione assay.

### The effect of combination treatments on γH2AX foci formation

**Figure 9:**
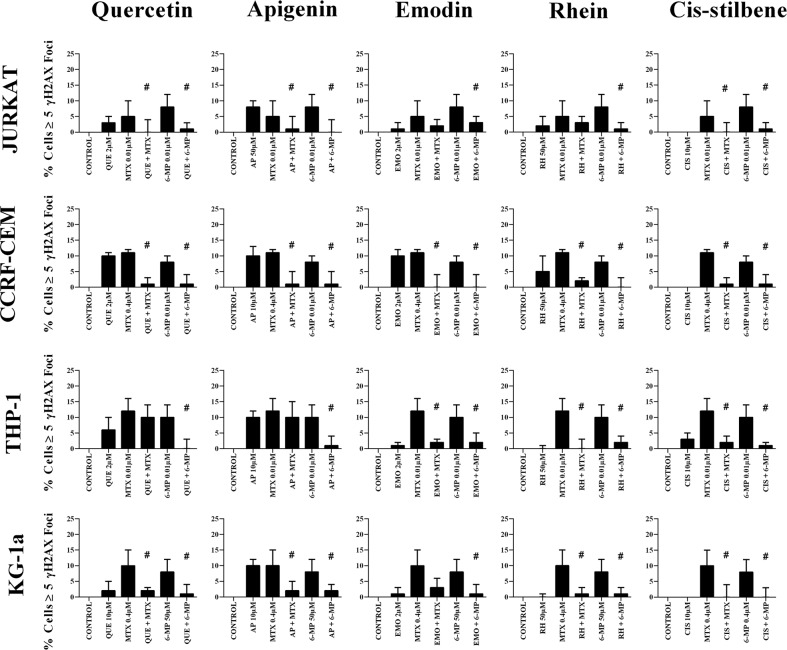
The effect of methotrexate (MTX) and 6-mercaptopurine (6-MP) when used in combination with quercetin (QUE), apigenin (AP), emodin (EMO), rhein, (RH) or cis-stilbene (CIS) on γ-H2AX foci formation (DNA damage marker): in two lymphoid (Jurkat and CCRF-CEM) and two myeloid (THP-1 and KG-1a) leukemia cell lines This was evaluated by the immunofluorescent staining using Alexa Fluor® 647 Mouse anti-H2AX (pS139). Cells were treated with MTX or 6-MP and polyphenols alone and in combination for 24 hrs using their lowest-significant doses (LSD). The data was expressed as medians and ranges (n=4). Data was normalised to the vehicle control which was assigned 0% of γ-H2AX foci formation (DNA damage marker). Effects of combination treatments were statistically classified as synergistic (^*^) causing an increase in the percentage of cells with γ-H2AX foci or antagonistic (^#^) causing an decrease in the percentage of cells with γ-H2AX foci; when compared to vehicle control, drugs alone and expected values of combination treatments. Statistical significant was set at P≤0.05.

Combination treatments with all studied polyphenols antagonized the formation of γ-H2AX foci induced by methotrexate and 6-mercaptopurine treatments alone, in all the leukemia cell lines (P≤0.05) (Figure 9).

## DISCUSSION

This study investigated the hypothesis that polyphenols could modulate the response of leukemia cells to the antimetabolite agents: 5-fluorouracil, methotrexate and 6-mercaptopurine, which are commonly used in the treatment of leukemia [1–6]. Methotrexate and 6-mercaptopurine antagonize folate acid metabolism resulting in inhibition of DNA and RNA synthesis and cell division [9]. Whilst 5-fluorouracil is an analogue of the RNA base uracil, and is a potential inhibitor of thymidylate synthase, a key enzyme required for the conversion of deoxyuridine monophosphate (dUMP) to deoxythymidine monophosphate (dTMP) within the folate acid cycle. This inhibition causes an imbalance of deoxynucleotides and increased levels of dUMP, which causes disruption of the folate cycle and results in DNA damage and cell death [26].

Here, we demonstrated that 5-fluorouracil combined with polyphenols induced differential effects which were dependant on the cell lineage, and the polyphenol used. Synergistic pro-apoptotic effects were observed following treatment with 5-flurouracil combined with quercetin, apigenin and rhein. These synergistic effects are consistent with work in other tumour types, where apigenin enhanced the activity of 5-fluorouracil in head and neck squamous cell carcinoma cell lines (SCC25) resulting in an increase in reactive oxygen species, correlating with a reduction in glutathione levels [27]. Likewise, quercetin enhanced 5-fluorouracil activity in colorectal (HCT116), prostate (PPC1) [18] and ovarian cell lines [28]. Furthermore, emodin enhanced 5-fluorouracil activity in non-small cell lung cancer cell lines [29].

In contrast, here 5-fluorouracil was antagonized by rhein in myeloid cells, and by *cis*-stilbene in all leukemia cells. Similarly, methotrexate and 6-mercaptopurine were antagonized by all polyphenols in the majority of leukemia cell lines tested. The exact mechanism of these antagonistic effects is currently unclear, however antagonistic effects correlated with increased glutathione levels and a reduction in DNA damage and apoptosis. Methotrexate and 6-mercaptopurine alone decreased ATP and glutathione levels, increased DNA damage, caspase 3 activity and apoptosis. These antifolates inhibit dihydrofolate reductase and tetrahydrofolate reductase, which subsequently impacts methionine metabolism and reduces glutathione levels [26]. However, these inhibitory effects were reversed by polyphenols, leading to the suggestion that polyphenols antagonize the antifolate effect of methotrexate and 6-mercaptopurine, resulting in folate and methionine metabolism continuing unabated, with the subsequent increase in glutathione being anti-apoptotic. It is well established that polyphenols act as antioxidants, but they can also have a pro-oxidant effect when used at high concentrations, or when used under certain experimental conditions [30–33]. The differential effect of polyphenols is suggestive of a specific drug pathway-polyphenol interaction, rather than a generalised pro-oxidant activity, which could deplete glutathione where synergistic responses are observed.

The structural similarity between antimetabolite agents and polyphenols and their metabolites may explain the antagonistic effects observed. Campo *et al*, (2009) suggested that polyphenols such as epigallocatechin gallate, epicatechin gallate, and quercetin-3-D-xyloside were structurally similar to methotrexate, and could inhibit dihydrofolate reductase activity within the folate cycle and hence inhibit DNA synthesis, and drive apoptosis [34]. This led to the suggestion that polyphenols could be used as antifolates and replace drugs such as methotrexate [34]. Therefore, the use of polyphenols in combination with antimetabolites such as methotrexate and 6-mercaptopurine; could competitively antagonize each other, as they target the same pathway.

Our previous work on polyphenol-induced sensitization to topoisomerase II inhibitors (doxorubicin and etoposide) showed that glutathione depletion predicted synergistic interactions and γ-H2AX [20–21], similar to effects when combined with 5-fluorouracil in this study. Whether depletion of glutathione and associated γ-H2AX foci is simply a proxy for apoptosis; or due to pro-oxidant polyphenol activity is unclear. However it is noteworthy that glutathione levels were increased where polyphenols antagonized methotrexate and 6-mercaptopurine, correlating with a lack of γ-H2AX foci, indicating the absence of DNA damage in both lymphoid and myeloid cell lines. Franco and Cidlowski (2009) reported high levels of glutathione were strongly correlated with over-expression of the anti-apoptotic protein Bcl-2, and the inhibition of mitochondrial-induced apoptosis [35]. This supports the notion that elevated glutathione levels oppose the pro-apoptotic chemotherapy responses [32, 35–37].

Interestingly, polyphenols have a biphasic effect on chemotherapy responses, whereby low levels of quercetin, in combination with 5-fluorouracil, cisplatin, taxol or pirarubicin induced an antagonistic effect inhibiting apoptosis, correlating with reduced intracellular ROS levels and increased expression of endogenous antioxidant enzymes, suggesting that low levels of quercetin could act as cytoprotective for cancer cells when combined with these chemotherapy drugs [28]. Conversely, higher doses (40-100 μM) induced synergistic responses which correlated with an elevation of intracellular ROS [28]. These findings are extremely important as they suggest that polyphenols commonly found in the diet have the potential to modulate anti-tumor activity of these antimetabolite agents, either acting synergistically enhancing their pro-apoptotic effects or more importantly, having an antagonistic effect inhibiting their function.

Previous studies suggest that the activity of chemotherapeutic agents can be enhanced by polyphenols [16–18, 20–21, 38]. However, importantly, polyphenols have also been shown to inhibit the response to chemotherapeutic agents in leukemia cell lines and other cancers [39–43]. It is noteworthy that quercetin and apigenin are readily detectable in serum following dietary intake in both humans [44], and rat studies [45]. Furthermore, pharmacokinetic studies have shown that dietary intake of polyphenols, including quercetin, resulted in a plasma concentration of 2.3 μg/ml [44]. This is equivalent to a 7.6 μM plasma concentration, which is comparable to the levels used in this study. Likewise, oral administration of apigenin has also been shown to alter the bioavailability of paclitaxel in rats, which demonstrates apigenin can alter systemic effects of chemotherapeutics [46]. Finally, plasma-levels of quercetin, derived from dietary sources, have also been shown to inhibit the efficacy of the proteasome inhibitor, bortezomib in chronic lymphocytic leukaemia cells [47]. Suggesting that the concentrations of polyphenols shown here, which were antagonistic to methotrexate and 6-mecaptopurine; are likely to be achievable via dietary sources alone [44]. These studies therefore demonstrate that polyphenols have the potential to both enhance and inhibit chemotherapy responses following dietary intake. Thus, it may be essential to monitor blood levels of polyphenols during chemotherapy treatments.

## MATERIALS AND METHODS

### Leukemia cell lines

Four human leukemia cell lines were used for this study: Two lymphoid leukemia cell lines (Jurkat (peripheral blood T cell leukemia) (ATCC: TIB-152, Middlesex, UK) and CCRF-CEM (acute lymphoblastic leukemia) (ATCC: CCL-119, Middlesex, UK)); which had been previously shown to be sensitivity to polyphenols treatments [15]; together with two myeloid leukemia cell lines (THP-1 (acute monocytic leukemia) (ATCC: TIB-202, Middlesex, UK) and KG-1a (acute myelogenous leukemia) (ATCC:CCL-243)) which had been previously shown to be resistance to polyphenol treatment [15]. Non-tumor control hematopoietic stem cells from cord blood (CD34^+^HSC and CD133^+^HSC) (Stem cell Technologies, Grenoble, France) were also included in the study. All cells were tested for mycoplasma contamination using the MycoAlert TM mycoplasma detection kit (Lonza, MD, USA) and were negative throughout the study.

### Culture conditions

Cells were maintained in Roswell Park Memorial Institute (RPMI) medium 1640 (Invitrogen, Paisley, UK) supplemented with 10% (v/v) fetal bovine serum, 1.5mM L-glutamine and 100μg/ml penicillin/streptomycin at 37°C with 5% CO_2_.

### Treatment regimes

Two lymphoid leukemia cell lines (Jurkat and CCRF-CEM), two myeloid leukemia cell lines (THP-1 and KG-1a); and two non-tumor control cells (CD34+HSC and CD133+HSC) were treated with each polyphenol and each antimetabolite agent: methotrexate, 6-mercaptopurine and 5-fluorouracil (Sigma, Poole, UK) alone or in combination, along with a vehicle control for 24 hrs. All treatments were performed in triplicate.

Quercetin (Enzo Life Sciences, Exeter, UK), apigenin, emodin, rhein and *cis*-stilbene (Sigma, Poole, UK) were prepared as described previously [15]. Methotrexate (MTX) and mercaptopurine (6-MP) were dissolved in 0.1M of NaOH alkylating solution; and 5-fluorouracil (5-FLU) was dissolved in water. Stock solutions (25mM) of each polyphenol were prepared with 10% (v/v) ethanol (Sigma) in serum free media (Invitrogen, Paisley, UK) to generate treatment concentrations between 0.005 and 50 μM. All polyphenols were HPLC analytical grade and were 90-97% pure. 10 μM NaOH and 0.1% ethanol vehicle controls were prepared for each treatment in all cell lines.

Dose response curves were generated for each antimetabolite agent. These were used to determine the IC_50_ dose (which statically reduced ATP levels by 50%) and lowest significant dose (LSD) (which statistically reduced ATP levels and induced caspase 3 activity when compared to the vehicle control at 24 hrs) (Table 1). Significance was determined using a Kruskal-Wallis with a Conover-Inman Post hoc-test. The LSDs for each polyphenol was determined in our previous study [15]. These LSD doses were used for subsequent polyphenol/chemotherapy combination work.

The LSDs determined from effects on ATP levels were used in combination studies investigating their effects on ATP levels and cell viability, cell-cycle progression, DNA damage and glutathione levels. Whilst the LSDs determined from the induction of caspase 3 activity, were used in combination studies investigating effects on apoptosis.

Measurements were made of cell ATP levels, cell-cycle progression and apoptosis in each leukemia cell line and non-tumour control cells following treatment with each polyphenol and antimetabolite agent alone and in combination. Subsequent measurements were made of glutathione levels and DNA damage in all cells following treatment with methotrexate or mercaptopurine alone or in combination each polyphenol.

### CellTiter-glo luminescent cell ATP viability assay

The CellTiter-Glo luminescent assay measure ATP levels and is indicative of cell viability and proliferation. Cells were seeded at 2.5 × 10^3^ cells per well of a white 96-well plates (Fisher Scientific, Loughborough, UK) and treated with each polyphenols and anti-metabolite agent alone and in combination for 24 hrs, together with a 0.1% (v/v) ethanol and a 10 μM NaOH vehicle control. Following treatments, the CellTiter-Glo luminescent cell viability assay (Promega) was used to measure ATP levels, as per manufacturer's instructions. Dose response curves were generated for each antimetabolite agent. These were used to determine the lowest significant dose (LSD) which statistically reduced ATP levels when compared to the vehicle control at 24 hrs (Table 1). These LSD for ATP were used for subsequent polyphenol/chemotherapy combination work on cell viability, cell-cycle progression, DNA damage and glutathione levels. The dose responses curves and calculations of LSDs for each polyphenol were determined in our previous study [15].

### Apoptosis analysis

#### Nucview caspase 3 activity assay by flow cytometry

Cells were seeded at 0.5 × 10^6^ cells per well in 12-well plates and treated with polyphenols and antimetabolite agents alone or in combination for 24 hr, together with a 0.1% (v/v) ethanol and a 10 μM NaOH vehicle control. Following treatments, the NucView caspase 3 activity assay (Cambridge Bioscience, Cambridge, UK) was used to measure caspase 3 activity as an indicator of early stage apoptosis as per manufacturer's instructions. Samples were analysed by flow cytometry as described previously [15]. Dose response curves were generated for each antimetabolite agent. These were used to determine the lowest significant dose (LSD) which statistically increased caspase 3 activity compared to the vehicle control at 24 hrs (Table 1). These LSD doses caspase 3 were used for subsequent polyphenol/chemotherapy combination treatments on the morphological assessment of apoptosis. The LSDs for caspase 3 activity for each polyphenol was determined in our previous study [15].

### Morphological assessment of apoptosis

The effects of the combination treatments of antimetabolite agents and polyphenols were further investigated on the apoptotic nuclear morphological changes following Hoechst 33342 and propidium iodide staining and fluorescence microscopy (Sigma). Cells were at 0.5 × 10^6^ cells seeded in 12-well plates per ml and treated for 24 hrs with each antimetabolite and polyphenol alone and in combination at their LSD. A 0.1% (v/v) ethanol and a 10 μM NaOH vehicle control were also included. Following 24 hrs of treatments, 100 μl of cells were transferred to a 96-well plate, 10 μl of 2μg/ml Hoechst 33342 dye was added to each well and incubated for 5 min in the dark. A further 10 μl of 2μg/ml propidium iodide dye was added and incubated for 15 min in the dark. Plates were examined using inverted fluorescence microscope. Two hundred cells (live and apoptotic) were counted and apoptotic nuclei determined for each sample and expressed as a percentage. The Cell-F software (Olympus) was used to capture fluorescent microscope images.

### Analysis of cell cycle progression

Cells were seeded at 0.5 × 10^6^ cells per ml in 12-well plates and treated with each antimetabolite agent and polyphenols alone or in combination at their LSD for 24 hrs, together with a 0.1% (v/v) ethanol and a 10 μM NaOH vehicle control. Following treatment cells were harvested and centrifuged at 400 g for 5 min. The supernatant was removed, and cells were washed twice in 100 μl cold PBS. Cells were fixed by adding 100 μl of 80% ethanol/H_2_O (v/v) and stored overnight at −20°C. Then, cells were washed twice with cold PBS prior to addition of 300 μl of 50 μg/mL PI (Sigma, Poole UK) and 50 μl of 0.1 unit/mL RNase (Sigma, Poole UK). Samples were PI stained overnight at 4°C and analyzed on the flow cytometer with BD FACS Calibur instrument. Ten thousand events were acquired per sample and the DNA histogram of cell cycle phase was analyzed with FlowJo software using the Waston (pragmatic) equation (Tree Star, Ashland, OR, USA).

### Measurement of cell GSH levels

Cells were seeded at 2.5 × 10^3^ cells per well into white 96-well plates and treated with methotrexate or6-mercaptopurine alone or in combination with each polyphenols at their LSD for 24 hrs, together with a vehicle control. Following treatment, glutathione levels were measured using the GSH-Glo glutathione luminescent assay as per manufacturer's instructions. Briefly, cell were washed in PBS and resuspended in 50 μl of PBS in a clean white 96-well plate (Fisher Scientific). Fifty microliters of GSH-Glo^TM^ reagent containing Luciferin-NT substrate and glutathione S-transferase (diluted 1:50 in GSH-Glo TM reaction buffer) (Promega) was added to each well. The content was gently mixed using a shaker for 2 minutes and then further incubated at room temperature for 15 minutes. The luminescent signal was measured using a Wallac Victor 2 1420 and normalised to the vehicle control.

### Measurement of DNA damage by γH2AX foci detection

The formation of γH2AX foci is indicative of DNA damage. The Alexa Fluor 647 Mouse anti-γH2AX (pS139) (BD Pharmingen, Oxford, UK) specifically targets phosphorylation of Ser-139 at the C-terminal region of γH2AX enabling the visualisation of γH2AX by immunofluorescence. Cells were seeded at 1.0 × 10^3^ cells per well into a BD Falcon 96-well imaging plate (BD Pharmingen) and treated with methotrexate and 6-mercaptopurine alone and in combination with each polyphenol at their LSD for 24 hrs, together with a vehicle control. Following treatments, cells were centrifuged at 400 × g for 10 min then washed in PBS and fixed in BD Cytofix fixation buffer for 10 min (BD Pharmingen). Cells were washed twice in PBS and permeabilized in 90% methanol (Sigma) for 5 min. Following washes, cells were incubated in 50 μl Alexa Fluor 647 Mouse anti-γH2AX (pS139) (1:10 v/v) at room temperature for 1 hr in the dark. Cells were washed 3 times in PBS and counter-stained in 100 μl of 2 mg/ml Hoechst 33342 stain for 15 min. Cells were visualised using inverted fluorescence microscopy (Olympus, 1 × 2-UCB). Cells with greater than six γ-H2AX foci were considered as having DNA damage. The number of cells with DNA damage (>6 foci) or without DNA damage (<6 foci) were counted. 200 cells per treatment were counted and percentage of cells with substantial DNA damage determined. Images were captured using an inverted fluorescence microscopy and the Cell-F software.

### Statistical analysis

The median with range was calculated for each assay. Stats Direct software (Stats Direct Ltd, Altrincham, UK) was used to test whether data followed a normal distribution using a Shapiro-Wilkes test, which was used to determine whether the data was parametric or non-parametric. As the data was non-parametric, a Kruskal-Wallis and Conover-Inman post-hoc test was used to determine statistical significance of the data. Results were considered statistically significant when P≤0.05.

The effect of combination chemotherapy and polyphenol treatment on ATP levels, apoptosis, glutathione levels and DNA damage were classified as: synergistic or antagonistic as described previously [15]. All results were considered statistically significant when P≤0.05.

Cell-cycle analysis was performed by determining the percentage of cells in each phase using the FlowJo software using the Watson pragmatic model. The data was expressed as medians with ranges (n=4). The statistical significance of individual drugs was determined firstly in comparison to the vehicle control using a Kruskal-Wallis and Conover Inman post hoc tests. The statistical significance of combined polyphenol and drugs treatments was determined in comparison to the vehicle control and the individual treatments alone. The effect of combination treatments on cell-cycle was classified either as: interactive, non-interactive or antagonistic using the method outline in Mahbub *et al* 2015. Statistical significance was set at P≤0.05.

## SUPPLEMENTARY MATERIALS FIGURES



## Abbreviations

MTX: Methotrexate
6-MP: 6-Mercaptopurine
5-FLU: 5-Fluorouracil
QUE: Quercetin
AP: Apigenin
EMO: Emodin
RH: Rhein
CIS: *Cis*-Stilbene
