# Supplementary material for: Dietary polyphenols influence antimetabolite agents: methotrexate, 6-mercaptopurine and 5-fluorouracil in leukemia cell lines
Source: Oncotarget. 2017 Aug 24;8(62):104877–93. doi: 10.18632/oncotarget.20501 (PMC5739607; doi:10.18632/oncotarget.20501)
Supplement: Supplementary file 1 [file oncotarget-08-104877-s001.pdf]

Dietary polyphenols influence antimetabolite agents: methotrexate, 6-mercaptopurine and 5-fluorouracil in leukemia cell lines

SUPPLEMENTARY MATERIALS

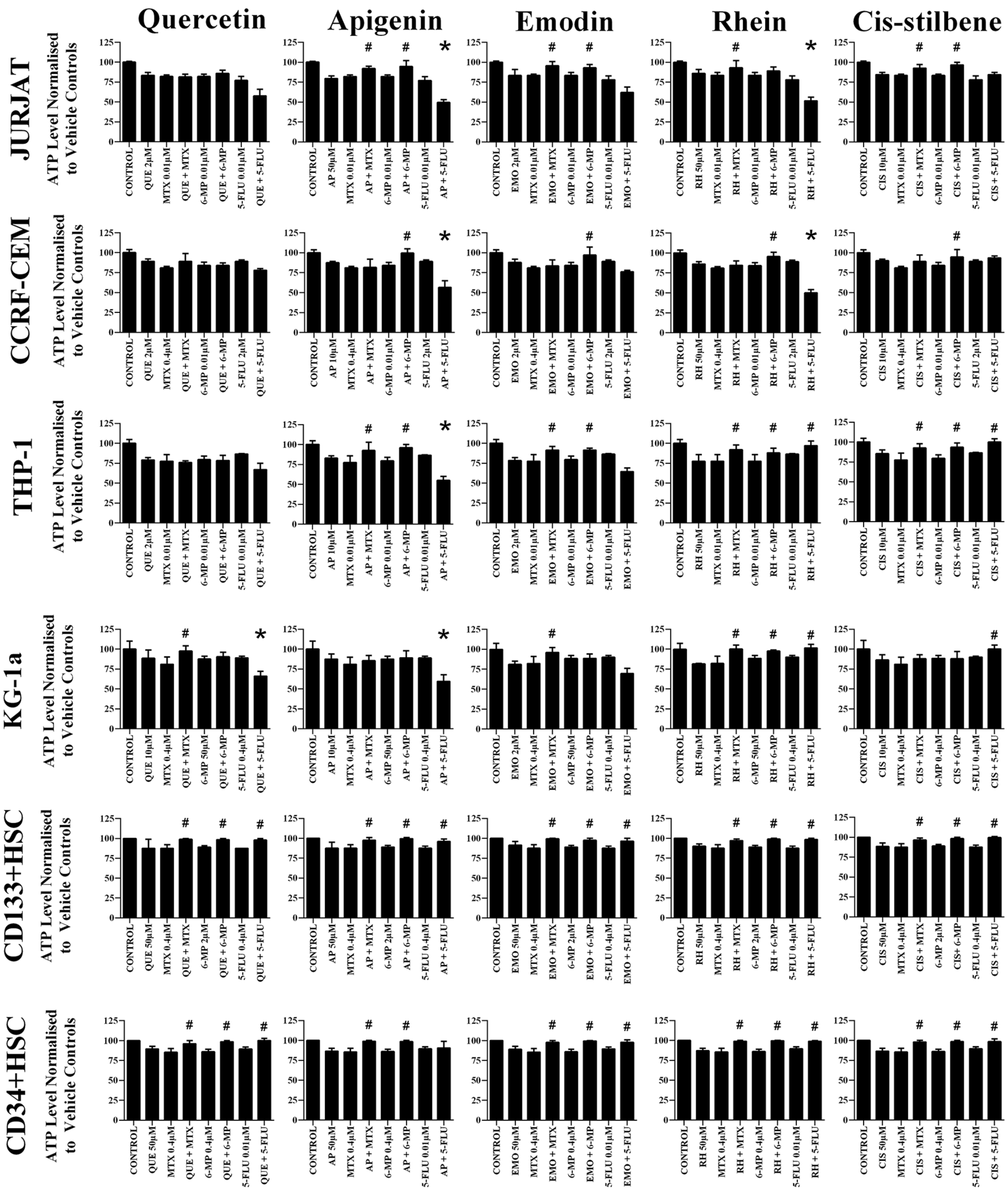

Supplementary Figure 1: The effect of methotrexate (MTX), 6-mercaptopurine (6-MP) and 5-fluorouracil (5-FLU) when used in combination with quercetin (QUE), apigenin (AP), emodin (EMO), rhein (RH), or cis-stilbene (CIS) on ATP levels: in two lymphoid (Jurkat and CCRF-CEM) and two myeloid (THP-1 and KG-1a) leukemia cell lines, and two non-tumor control cells ($CD133^{+}$ HSC and $CD34^{+}$ HSC). This was evaluated by CellTiter-Glo® assay. Cells were treated with MTX, 6-MP or 5-FLU and polyphenols alone and in combination for 24 hr using their lowest-significant doses (LSD); together with a vehicle control. All data was normalised to the vehicle control which was assigned 100% cell viability. The data was expressed as medians and ranges (n=4). Effects of combination treatments were statistically classified as synergistic (*) causing a decrease in ATP levels or antagonistic (#) causing an increase in ATP levels; when compared to vehicle control, drugs alone and expected values of combination treatments. Statistical significant was set at P≤0.05.

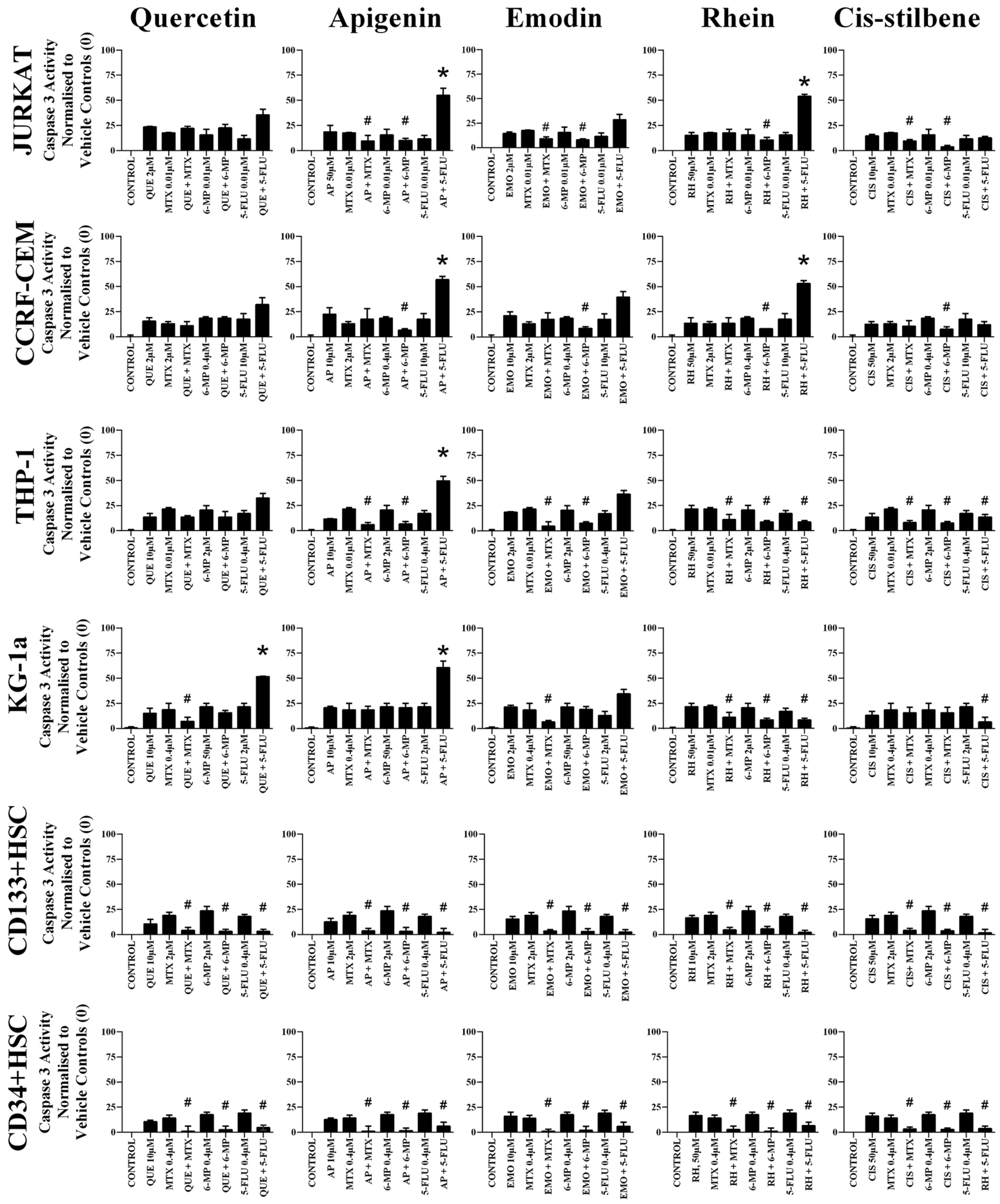

Supplementary Figure 2: The effect of methotrexate (MTX), 6-mercaptopurine (6-MP) and 5-fluorouracil (5-FLU) when used in combination with quercetin (QUE), apigenin (AP), emodin (EMO), rhein (RH), or cis-stilbene (CIS) on caspase 3 activity: in two lymphoid (Jurkat and CCRF-CEM) and two myeloid (THP-1 and KG-1a) leukemia cell lines, and two non-tumor control cells ($CD133^+$ HSC and $CD34^+$ HSC). This was evaluated by NucView caspase 3 activity assay. Cells were treated with MTX, 6-MP or 5-FLU and polyphenols alone and in combination for 24 hrs using their lowest-significant doses (LSD), together with a vehicle control. All data was normalised to the vehicle control, which was assigned 0% apoptosis. The data was expressed as medians with ranges (n=4). Effects of combination treatments were statistically classified as synergistic (*) causing an increase in caspase 3 activity or antagonistic (#) causing an decrease in caspase 3 activity; when compared to vehicle control, drugs alone and expected values of combination treatments. Statistical significant was set at P≤0.05.

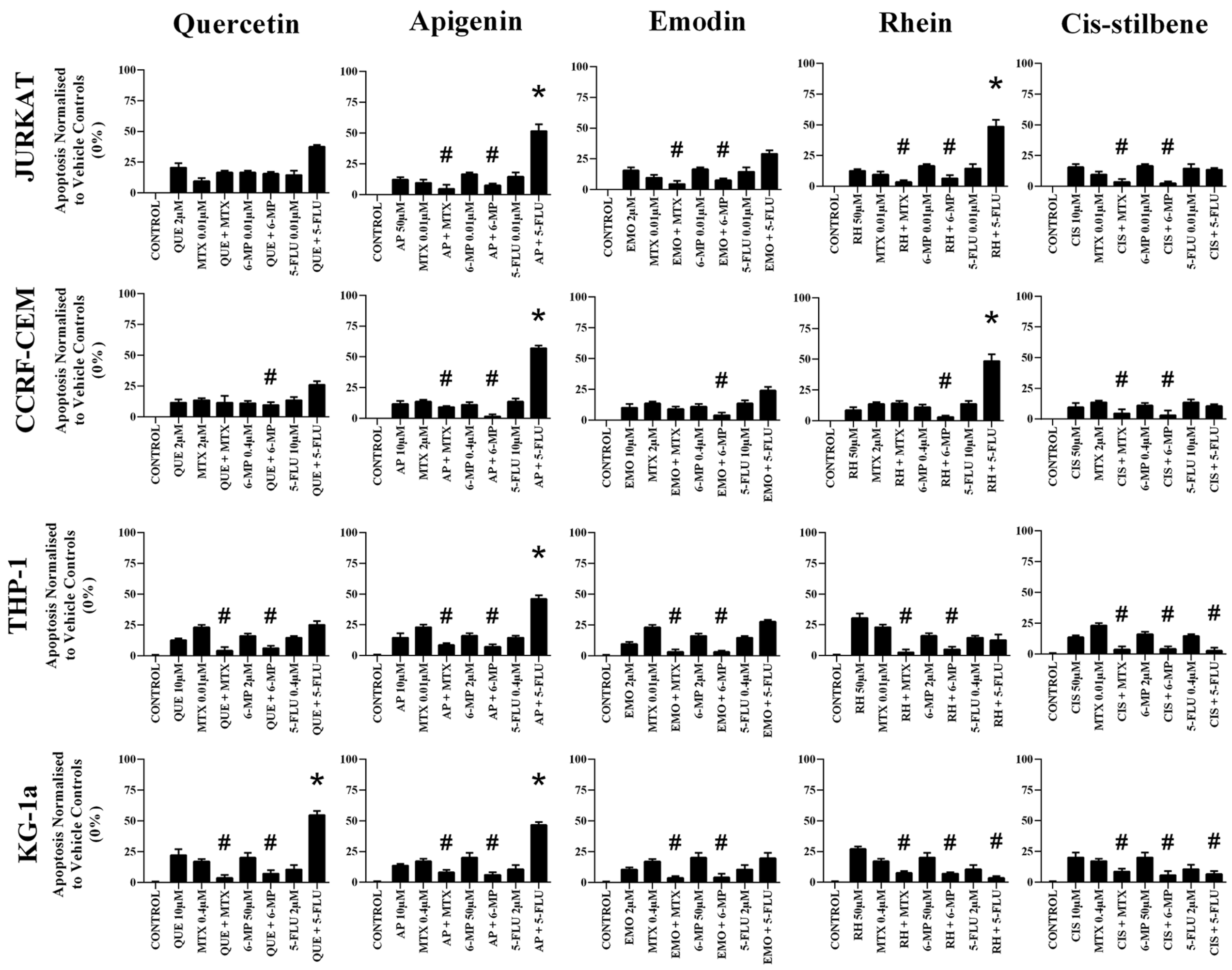

Supplementary Figure 3: The effect of methotrexate (MTX), 6-mercaptopurine (6-MP) and 5-fluorouracil (5-FLU) when used in combination with quercetin (QUE), apigenin (AP), emodin (EMO), rhein, (RH) or cis-stilbene (CIS) on apoptosis morphological changes: in two lymphoid (Jurkat and CCRF-CEM) and two myeloid (THP-1 and KG-1a) leukemia cell lines. This was evaluated by double staining with Hoechst 33342/PI using fluorescence microscopy. Cells were treated with MTX, 6-MP or 5-FLU and polyphenols alone and in combination for 24 hrs using their lowest-significant doses (LSD). Data was normalised to the vehicle control, which was assigned 0% apoptosis. The data was expressed as medians with ranges (n=4). Effects of combination treatments were statistically classified as synergistic (*) causing an increase in apoptosis or antagonistic (#) causing a decrease in apoptosis; when compared to vehicle control, drugs alone and expected values of combination treatments. Statistical significant was set at P≤0.05.

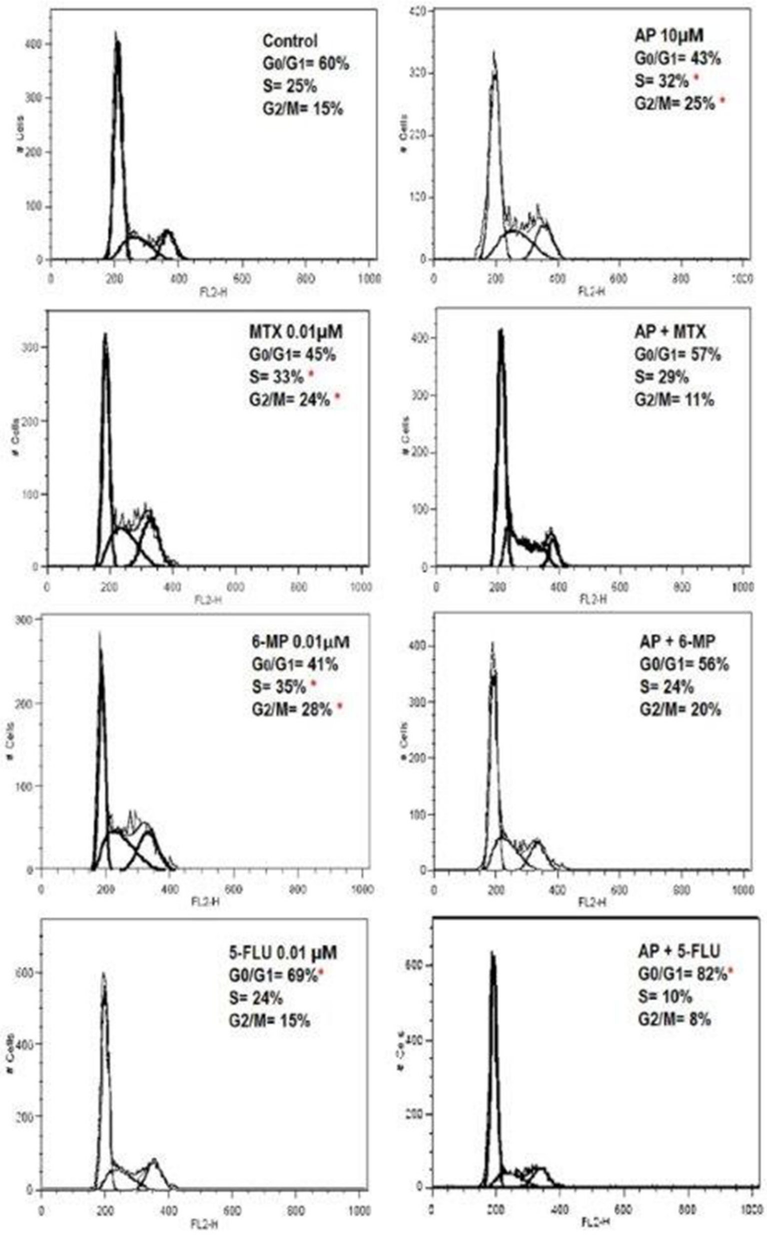

Supplementary Figure 4: An example of histograms showing the distribution of cells in the cell cycle phases (G_0/G_1, S, G_2/M) following methotrexate (MTX), 6-mercaptopurine (6-MP) and 5-fluorouracil (5-FLU) treatment alone, and in combination with apigenin (AP) at their LSD doses for 24 hrs in THP-1 myeloid leukemia cells. * indicates a statistically significant increase in cells accumulating within a phase of the cell cycle. Statistical significant was set at P≤0.05.

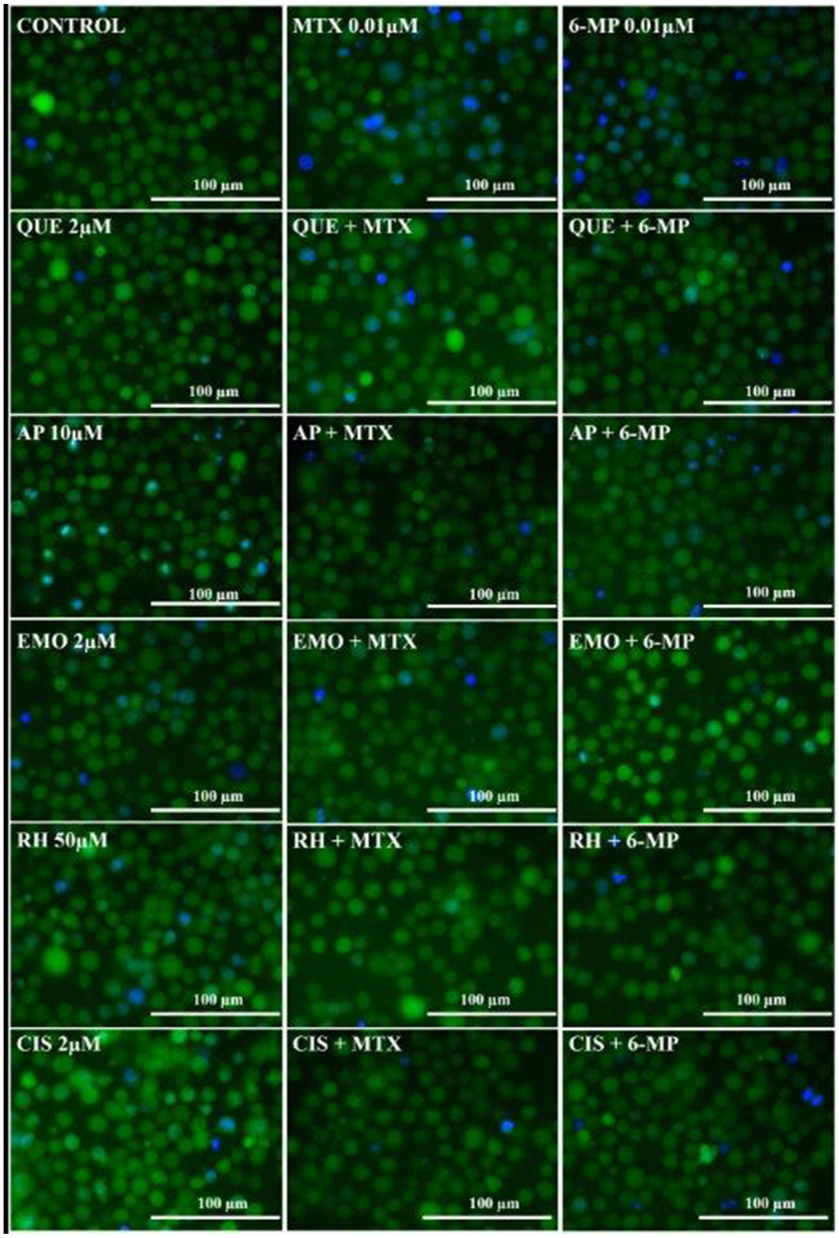

Supplementary Figure 5: An example of glutathione (GSH) levels in THP-1 myeloid leukemia cells when treated with LSDs of methotrexate (MTX) and 6-mercaptopurine (6-MP) and polyphenols: (quercetin (QUE), apigenin (AP), emodin (EMO), rhein, (RH) or cis-stilbene (CIS)) alone and in combination for 24 hrs. Cells with green-CMFDA staining indicate the presence of GSH localised in the cell cytoplasm and nucleus of live cells; whilst cells with blue Hoechst 33342 staining indicate a decrease in GSH localised in the nuclei of both live and dead cells. Scale bar = 100 µm.
